# Job Scheduling with Efficient Resource Monitoring in Cloud Datacenter

**DOI:** 10.1155/2015/983018

**Published:** 2015-09-15

**Authors:** Shyamala Loganathan, Saswati Mukherjee

**Affiliations:** Department of Information Science and Technology, Anna University, Tamil Nadu 600025, India

## Abstract

Cloud computing is an on-demand computing model, which uses virtualization technology to provide cloud resources to users in the form of virtual machines through internet. Being an adaptable technology, cloud computing is an excellent alternative for organizations for forming their own private cloud. Since the resources are limited in these private clouds maximizing the utilization of resources and giving the guaranteed service for the user are the ultimate goal. For that, efficient scheduling is needed. This research reports on an efficient data structure for resource management and resource scheduling technique in a private cloud environment and discusses a cloud model. The proposed scheduling algorithm considers the types of jobs and the resource availability in its scheduling decision. Finally, we conducted simulations using CloudSim and compared our algorithm with other existing methods, like V-MCT and priority scheduling algorithms.

## 1. Introduction

Cloud computing is an innovative, cost-effective delivery model [1], which is fast becoming an adaptable technology for many of the organizations with its dynamic scalability and usage of virtualized resources as a service through the Internet. In IaaS cloud, the resources (compute capacity and storage) are provided in the form of virtual machines to users. The objective is to deliver virtual servers having a predefined configuration [1]. An additional objective is to minimize underutilizations of the underlying infrastructure ensuring effective efficiency. To this end, resource management and scheduling are the required mechanism. The primary challenge of scheduling in a cloud environment is the allocation of available resources effectively thereby improving efficiency of the whole cloud computing environment. Presently, most of the cloud providers rely on simple resource allocation policies like immediate and best effort [2]. Though advanced reservation technique is a well-studied phenomenon in grid environment, immediate and best effort provisioning is preferred in public clouds owing to the dynamic nature of incoming request. Primary reason for the absence of predictability or a predefined usage pattern of cloud requests is its dynamic nature. Hence advanced reservation technique continues to be restricted in grid and is considered to be not appropriate for public cloud. However, this scenario is a little different for organizational cloud (private cloud) where it is possible to predict a usage pattern at least to an extent. This is perhaps due to the fact that a private cloud is owned and maintained by an organization and is typically used by the employee and other people related to that organization. This is the reason, we argue, that in private cloud with predictable resource usage, scheduling with different policy will enhance the resource utilization and ensure the guaranteed service. In general resource requirements of a system depend on the nature and specification of applications. The requests for computational resources can be of three types [3].

- Advance reservation (AR): resources are reserved in advance for this type of jobs. Resources are expected to be available at the specified time, when the job arrives.
- Immediate (IM): when a client submits a request, based on the resource availability, either the required resources are provisioned immediately or the request is rejected.
- Best effort (BE): these jobs, on their arrival, are provided with the required resources if available; else these are queued for the fulfillment of their requirements when the resources would be available. These can be batch jobs.

Incoming job requests can be categorized into these three types and scheduling the requests can be prioritized accordingly. Typically, BE jobs are not dead line sensitive requests and hence these can be queued to utilize underutilized or idle resources only. This approach will maximize resource utilization and provide a greater guarantee of servicing of incoming requests [3]. Job requests are submitted in the form of virtual machines. Typical attributes of virtual machine components are the number of cores required, CPU, memory, and bandwidth of a system needed to execute the job. In this paper, we propose an efficient scheduling algorithm to achieve high throughput and greater utilization of resources. The number of cores and the amount of memory given in the job request are considered as the capacity requirement of a VM in the proposed scheduling algorithm and we claim that other attributes may be easily incorporated in the algorithm. Many research efforts emphasize the need for exploring avenues to dynamic resource scheduling approaches in cloud. We propose a scheduling algorithm based on the earliest availability of resources with job requirement specification and a new data structure for monitoring resource availability efficiently. In the proposed system our contributions are as follows:

- improved cloud architecture with an efficient data structure for resource monitoring and lookup mechanism,
- a new preemption aware scheduling policy based on the types of jobs where starvation of the preemptable BE requests is avoided through flag indication,
- an efficient heuristic algorithm with modified best fit with capacity based scheduling in a datacenter that maximizes the resource utilization.

The remainder of this paper is organized as follows. We give a brief related work in Section 2. Next, in Section 3 we present our system model and Section 4 gives the problem formulation of the proposed algorithm.  Section 5 gives the evaluation of results and analysis of our work with two existing works.  Section 6 brings the rear with conclusion.

## 2. Related Work

A significant amount of research is focused on scheduling in grid environment where the advanced reservation technique is well studied. Algorithms proposed in [4, 5] discussed advance reservation and nonpreemptive task scheduling in a grid environment. Mapping of AR request with the available resources is proposed in these works. Sulistio et al. [6] proposed a data structure called GarQ specially for keeping advanced reservation requests and computing nodes available which is distributed in different locations as a grid. In our work, this data structure is explored in order to be used in cloud with different perspective.

In cloud computing, many works were explored with the aim of reducing rejection rate of request, maximizing profit, and improving the resource utilization. To achieve these goals, the scheduling algorithms are mainly focused on the request grouping or resource availability mapping by applying heuristic techniques. Selvarani and Sadhasivam [7] proposed an improved cost-based scheduling algorithm for making efficient mapping of jobs to the available resources by grouping the jobs based on maximum profitable jobs. The focus of this algorithm is on provider's profit rather on user satisfaction. Li et al. [8] proposed a feedback preemptible task scheduling algorithm to generate scheduling with the shortest average execution time of jobs. However the proposed algorithm may lead to starvation since jobs with longer execution time are kept in the queue.

In [9], Yang et al. presented V-MCT, a V-heuristics for job allocation, which allocates every job in an arbitrary order of minimum completion time of the virtualized resource. In this algorithm, only the completion time of the VM is considered but not its resource capacity. In this approach, VMs of different hosts are scheduled which reduces the resource utilization. Ghanbari and Othman [10] presented a priority job scheduling in cloud computing by using statistical method. Each job requests a resource with predetermined priority. A comparison matrix of each job according to resources accessibilities is computed. For each of the comparison matrices, priority vectors (vector of weights) are computed. Based on these priority vectors, resources are allocated. This algorithm has drawback in complexity, consistency, and finish time. A multiqueue scheduling (MQS) algorithm [11] is proposed to reduce the cost of both reservation and on-demand plans using global scheduler. The proposed methodology depicts the concept of clustering the jobs based on burst time. Jobs are sorted based on the ascending order of the burst time and are assigned to three different queues small, medium, and long based on the burst time of the jobs. Equal weightage is given to all the queues to schedule the job based on first come first serve. However, users' preferences are not considered. Also an important job with less burst time may be queued for long time. This may lead to an unpredictable execution time for user's request.

Abu Sharkh et al. [12] proposed a scheduling algorithm for advanced reservation requests where he considered the available resources and software defined networks (SDN) for allocation. He proposed a greedy algorithm of allocating start time first and has shown that the tardiness is minimized by the proposed algorithm. In their algorithm, they considered only the public cloud on demand resource pattern and limited the scheduling to advanced reservation type of request only. Kaushik et al. [13] proposed a flexible reservation window scheme. It concludes that when the size of the reservation window is equal to the average waiting time in the on-demand AR queue, the reservation rejection rate can be minimized close to zero. But the work considers only advance reservation requests and hence does not address the issue of low resource utilization. All the abovementioned works have considered only advanced reservation requests but not the other request types. kurdi et al. [14] proposed an antistarvation algorithm to avoid BE jobs to be queued for long. This algorithm rejects some of the AR requests to accommodate BE requests or forcefully converted some AR requests as BE request. The main disadvantage of this algorithm is the absence of mechanisms for user satisfaction. Nathani et al. [15] proposed a scheduling algorithm that supports four kinds of resource allocation policies: immediate (IM), best effort (BE), advanced reservation (AR), and deadline sensitive (DS). The requests are termed leases. On the arrival of a request, scheduler tries to schedule the request in a single slot or multiple slots. If it is not possible, then it tries to schedule the request by modifying the existing schedule by using swapping and backfilling (SAB) techniques. In swapping, two consecutive leases are swapped if and only if the first lease has requested fewer resources than second lease and after swapping their deadlines are not missed. Backfilling procedure is applied to schedule BE and DS leases by rescheduling them into multiple idle slots. SAB techniques are applied to push idle resources towards requested time slot of a new lease. In this method, swapping of BE requests with DS leads to starvation since there is no indication of how many times it gets swapped.

Inspired by these works, we consider the job type and capacity based preemption technique where the starvation of the BE request is eliminated through the proposed resource monitoring mechanism. Further, the idle resources are identified efficiently and scheduled using the proposed scheduling algorithm to improve resource utilization.

## 3. Proposed System Model

The following are adaptations/assumptions in the proposed model.

1. Jobs are classified into three types as advanced reservation, immediate, and best effort where advanced reservation and immediate can preempt the best effort jobs and they are not preemptible.
2. The best effort jobs are backfilled and maintained by the Control Management System (CMS) to be scheduled when the resources are free.
3. Though the computing resource means core, memory, and bandwidth, we consider core and memory as resource capacity with the assumption that the bandwidth is more or less the same in private cloud.

**Figure 1:**
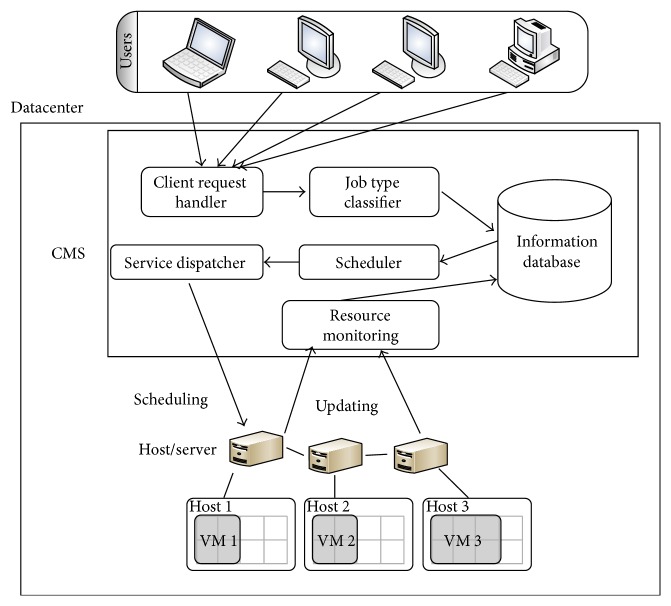
System architecture of the proposed model.

In a cloud, the end users' service requests are considered as job and the job is assigned to a virtual machine (VM). In the proposed model, the hosts are assumed to be homogeneous physical machines (or servers) that contain the computational power where the VMs are deployed. Since the proposed algorithm focuses on the available cores and memory, adaptation of the proposed model in a heterogeneous environment is a straight forward task. The architecture of the proposed cloud model is shown in Figure 1 and notations used in the system model are described in Notations Section.

The proposed model pivots around a central mechanism named CMS (Control Management System) and a datacenter that consists of *m* homogeneous hosts (servers) is interconnected with the CMS and there may be a total of *J* jobs in the system. Typically, CMS is a centralized server controlling all the hosts present in the datacenter and has a web portal for job submission, request and service handler, resource monitor, scheduler, and necessary databases. In the proposed model, CMS has additional components job type classifier and modified resource monitoring with specific data structure which is explained in detail in the next subsection.

Clients submit their jobs to the CMS using the portal and these incoming jobs are kept in the request queue RQ maintained by the CMS. We follow the mechanism used in Amazon EC2 and have VMs with four different sizes, namely, small, medium, large, and X-large depending on the number of cores. Each host consists of *x* number of cores which are assigned to the VMs based on the VM type. Job requests are assigned to these VMs and can execute in parallel on a host with different finish time. For each host, the proposed CMS maintains a job queue JQ_*t*_, where the jobs assigned by the CMS to that host are queued.

On receiving job request, CMS imposes job type as advanced reservation, best effort, and immediate garnered from the information contained in the request. The best-effort jobs do not have any time constraints, such as start and end times. Immediate and advance reservations jobs come with specific time constraints. CMS will preempt the best-effort job whenever the resources are required for immediate or advanced reservation job request. Apart from that, CMS is responsible for scheduling the incoming jobs to the host. To identify a suitable host, the CMS employs the proposed modified best fit with capacity based scheduling (MBFCBS) algorithm discussed in Section 4. The components of the proposed CMS are described below.

### 3.1. Client Request Handler

Client request handler presents a GUI for job submission. It receives the incoming requests from the user and sends the requests to the job type classifier to identify the job type.

### 3.2. Job Type Classifier

Job type classifier in CMS helps to classify the incoming requests into three different types. Generally a request consists of a tuple <Num_core, a_Ram, a_D, BW, Exe_time, and St_time, End_time>, where Num_core is number of cores required, a_Ram is memory in megabytes, a_D is disk space in megabytes, BW is the network bandwidth in megabytes per second, Exe_time is the execution time, St_time is start time, and End_time is finish time (timestamp contains date and time). Since these are distinct requirements of each job, the request tuple can be used to identify the type of a submitted job as follows.

To identify the type of the job, a request is described as

 AR Job request = <Num_core, a_Ram, a_D, BW, Exe_time, St_time, End_time>
 BE Job request = <Num_core, a_Ram, a_D, BW, Exe_time, Nil, Nil>
 IM Job request = <Num_core, a_Ram, a_D, BW, Exe_time, St_time, Nil>

They are labeled and kept in request list RQ to be scheduled by the CMS.

### 3.3. Resource Monitoring

**Figure 2:**
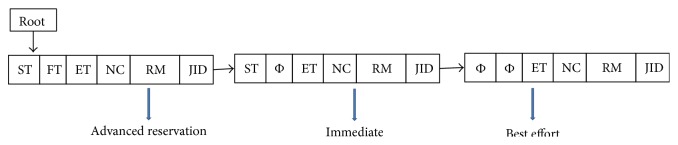
Representation of data structure as linked list of a host.

This component monitors and gathers information of a host such as running job (VM), number of executing cores, free core availability, and assigned AR jobs. This component prepares a resource availability list (RAL) and a preemption List (PL) from the information it got from the hosts. Essentially the RAL contains list of tuples <Host ID, CurrentTime, Free core availability, Free memory availability, Earliest Core available, Earliest Available Time (EAT)> and PL contains <Host ID, Job ID, Number of cores assigned, Time interval, Flag status>. Earliest available time (EAT) can be calculated using (1) and (3) below. To prepare the list, the component calculates the EAT of a core and the number of available cores for a time interval and updates the list whenever a new job is assigned to a host or when a job completes releasing resources. We observe that CMS needs to refer to and use the information about the available resources from RAL and PL. Hence it is important that these lists are kept updated. We propose to use an appropriate data structure using which the two lists can be updated without delay. The proposed data structure aids in the search, retrieval, and updating mechanisms that take place.  Figure 2 shows the modified partial data structure as linked list [16] is used in this research, which accommodates different job types. We identify the following basic operations to be performed by the data structure:

- search: checking for whether a core is free or available in a given time interval,
- add: inserting a new reservation request into the data structure,
- interval search: searching for the next available start time within a given time interval,
- job search: searching for BE mode job in a host for preemption within a given time interval.

To achieve the abovementioned characteristics, we propose to use a grid data structure inspired by GarQ [6], which is the combination of Calender Q [17] and array for advanced reservation requests. In order to support different job types in the proposed system, the data structure has been modified to consider the job types as a tuple represented as <ST, FT, ET, NC, RM, JID> where ST is start time, FT is finish time, ET execution time, NC is number of cores requested, RM is amount of memory required, and JID is job ID in a linked list as shown in Figure 2.

**Figure 3:**
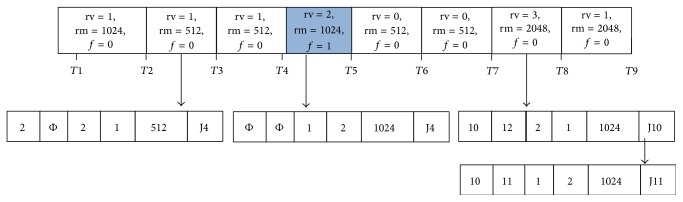
Representation of job allocation with time slot.

The proposed data structure has buckets with fixed smallest slot duration *α*, as with the calendar queue. Let *rv* be a variable which takes the value of number of cores in use or assigned and *rm* is the amount of memory allotted for a job request. Each bucket in the Calender Q contains *rv* value, *rm* value, flag for job type identification, and a sorted linked list containing the requests in the time bucket as shown in Figure 3. This approach makes the search operation for preparing RAL and PL easier since it only searches for a list inside each bucket. For searching available cores, require *O*(*k* *m*_sub_), where *k* is the number of requests and *m*_sub_ is the number of buckets within a subinterval [16]. The search operation and the advantages of the data structure are given in detail in [6]. Flag is set as 1 if it is a BE request and as zero for advanced reservation and immediate requests. In Figure 3, the BE request assigned is represented as shaded portion. In order to avoid the starvation of BE job by preempting several times, it is restricted through the flag. The flag is set to 2 if the preempted BE job is assigned again to the host when it is free. The PL is updated if the flag becomes 2 for that jobID, and then the job will not be taken into consideration for preemption again. From this detail, the resource monitoring component finds EAT of a host by calculating the availability of its cores and finish time from the data structure and updates the RAL whenever a new job is assigned to it. The EAT of a host is calculated using (1) and (3).

If the host is not full and some of the cores and memory are freely available, then they are calculated using (1). Let rA be free cores available in a host without assigning any job; AT is available time, and then EAT of a free core is calculated as

(1) EATrAt,rMt,ATt=Ct−rvt,Memt−rmt,CTHtwhere  t=1,m,

where rv_*t*_ is scheduled cores and rm_*t*_ is the scheduled memory of host *t*. CT(*H*_*t*_) is the current time of the host. rv_*t*_, rm_*t*_ are calculated from the data structure as

(2) rv∑NCji  over a time slot  T  and  i=1,J,rm∑RMji  over a time slot  T  and  i=1,J.

If none of the cores of a host is available in that time slot, we need to calculate the availability of a host by considering each job's finish time in the job queue of the host for the specified time interval *T*. Let a job *j*_*i*_ be in *t*th host with NC cores, RM memory, start time ST, execution time ET, and finish time FT in a time interval *T*, and then

(3) EATrAt,rMt,ATt=NCjit,RMjit,FTjit+εwhere  t=1,m,  i=1,J,

where NC(*j*_*i*_)_*t*_ is the number of cores, RM(*j*_*i*_)_*t*_ is the amount of memory used by the job *j*_*i*_, and FT(*j*_*i*_)_*t*_ is the finish time of the job where *ε* is a small slack value for delay for next job to start. Using these equations the RAL is prepared. The RAL and PL are modeled as indexed B tree with sorted order [18] which has the search operation in *O*(log⁡*m*) which is an efficient data structure for range queries. The lists are prepared offline and updated whenever changes occur.

### 3.4. Scheduler

Scheduler component in CMS finds a suitable host for an incoming request and assigns the request to that host for execution. The scheduler gets the RAL and PL from the database and applies (4) to find the host to assign the job:

(4) EATHt=availrAt, availrMt,min⁡ATjit∀jit∈JQt  i=1,J,  t=1,m.

EAT(*H*_*t*_) is the host with available number of cores and available amount of memory with the minimum finish time, so that it can be scheduled next if the capacity requirement of the incoming job request is satisfied.

### 3.5. Service Dispatcher

The service dispatcher dispatches the job request to the corresponding host for execution.

### 3.6. Information Registry

The incoming requests in RQ, RAL, and PL are maintained in the database which gets updated whenever new jobs are assigned to a host.

## 4. Problem Formulation

The problem of job scheduling in a cloud environment essentially consists of a dynamic set of *J* independent job requests to be scheduled on a set of m computational nodes in a datacenter. The resources in the cloud system are requested in terms of VMs which is nothing but the job request. The resources in the cloud system are utilized in terms of cores in a host where a host contains several cores, and a hence host can be utilized by several jobs at the same time. Hence we argue that assigning multiple jobs (VMs) on the same host is a bin packing problem [19] and can be represented as integer linear programming model.

### 4.1. Mathematical Model

In this paper, we deal with job scheduling problem of *m* physical machines. Physical machine *H*_*t*_ can allocate at most *n* jobs (virtual machines) at any time. Each user job *j*_*i*_ in the system could demand a service which needs *x* cores (NC) and *y* amount memory (RM) for a VM *v*_*k*_. All the variables and constants used in the model are listed for easy reference as follows:

(i) *n* is the number of VMs in a host utilizing rv cores and rm memory using (2);
(ii) *m* is the number of hosts/servers in the datacenter;
(iii) *X* is a binary variable indicating that VM *v*_*k*_ is assigned to a server *t*;
(iv) *Z*_*t*_ is a variable used to indicate whether the server *t* is used or not.

The proposed job scheduling algorithm is an extended bin-packing approach with the constraints or inequalities. The objective is to pack items (VMs) into a set of bins (servers or host hosting the VMs) characterized by their availability. At run time, each server *t* hosting a number of VMs is characterized by its remaining capacity (free cores and free memory) and earliest availability of cores and memory if no cores and memory are available for the next execution. Since the objective is to pack maximum number of VMs on a host for executing job requests, the constraints are given as follows.

(1) A VM represents only one job request at a time.
(2) Each server has its capacity limit CP_limit_.
(3) Each requested VM is assigned to one server
  (5) $$\begin{array}{c} \overset{m}{\underset{t=1}{\sum }}X_{tk}=1\;\forall k=\left[1,n\right]. \end{array}$$
(4) At any time, total number of cores and memory required by the virtual machines in physical machine (*H*_*t*_) does not exceed its capacity (CP_limit_). Let *c*_*k*_ denote the capacity (number of cores and memory) required by a VM and then
  (6) ∑k=1nckXtk≤CPlimit∀t=1,mwhere  ck=NCvk&&RMvk.
(5) The maximum utilized host is chosen to increase the resource utilization provided that the requested capacity is available. *H*(*C*_max⁡_) denotes the host with maximum capacity utilized.

Job scheduling model can be summarized by the objective function with all the constraints and conditions as

(7) Minimize H=∑t=1mHCmax∗Zt,

(8) Subject To ∑k=1nckXtk≤CPlimit∀t=1,m,

(9)  ∑t=1mXtk=1∀k=1,n,

(10)  Zt=1,if the server  t  is used,0,otherwise,

(11)  Xtk=1,if the VMk placed in Server  t,0,otherwise,

(12)  HCmax⁡t≥cki∀t=1,m,  k=1,n,  i=1,J.

Since the solution to this is NP-complete [20], a greedy set heuristic algorithm can be used to get the suboptimal solution to the global optimum. We used the best fit heuristic algorithm with the modification of capacity based scheduling to optimize the resource utilization by minimizing the number of host.

### 4.2. Job Scheduling in Datacenter

The objective of the scheduler is to maximize the resource utilization by allocating earliest available hosts by reducing the number of hosts. To maximize the resource utilization, we have proposed the modified best fit with capacity based scheduling (MBFCBS) algorithm which is shown in Algorithm 1. Since AR/IM jobs can preempt BE jobs, the only case where an AR/IM job is rejected is that most of the resources are reserved by some other AR at the required time, and insufficient resources are left for this job. If there are *n* numbers of VMs executing on a host with different finish time, then a new request is scheduled on the same host provided that the capacity of the finishing VM is greater than or equal to the new requested VM.

The algorithm allocates the VM to a host provided that the required capacity is satisfied by the available resource capacity in the host. A host may have multiple BE requests scheduled in a time interval *T*. During preemption, a BE request, which first fits the capacity requirements and satisfies the start time of the incoming request is chosen for preemption. In order to avoid multiple preemptions of the same BE job, flag is used while preempting a BE job request.

## 5. Experiments and Evaluation

In this section we present an evaluation for our algorithm in terms of performance with respect to certain performance metrics, appropriate workloads, and the simulation environment. We have used two setups: a small real-time setup to evaluate the performance of the proposed algorithm in a real time environment and a simulated setup to check the performance of the proposed algorithm in a larger environment.

### 5.1. Real-Time Experiment Setup

**Figure 4:**
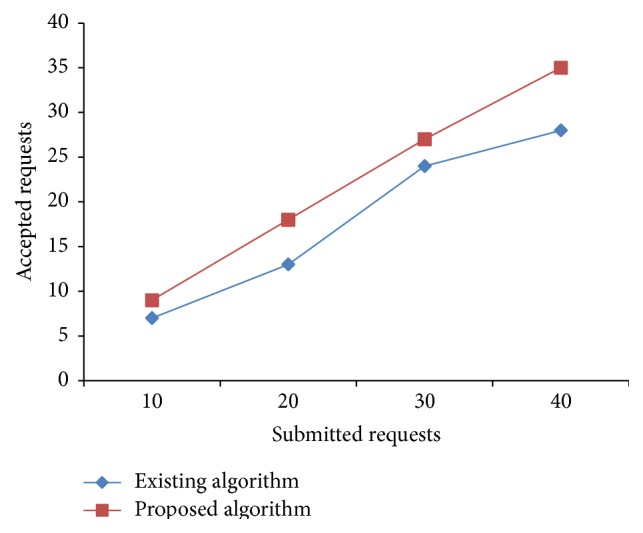
Scheduler comparison of Haizea-accepted requests.

**Figure 5:**
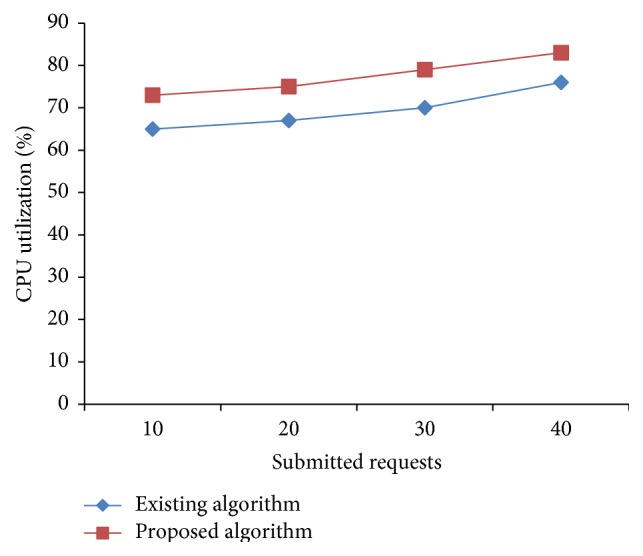
Utilization comparison of Haizea.

Haizea [21] VM scheduler is used to evaluate the proposed algorithm in real time in a small cluster. Haizea's VM scheduler component is modified to implement the algorithm. Code is written in python and run to evaluate our model. We used four physical nodes each having 4 cores and 1 GB memory attached which is considered as the available resources in provider side. The experiment is performed by considering four sets of 10, 20, 30, and 40 leases, respectively. Each of the input parameters in all four sets follows random distribution with the mix of three types of requests. The submitted number of requests of different sizes is varied and readings are taken for the number of requests accepted. The CPU utilization is calculated as the number of requests allocated and executed on a host to its total capacity. Haizea allocates requests in the form of leases. It supports advanced reservation lease. The default algorithm of Haizea applies greedy method of choosing VM and assigns the request. The proposed algorithm applies the concepts of preemption and EAT (VM) which gives more assignment in addition to the methods used by existing algorithm. Allocation based on our algorithm resulted in improved request acceptance rate, reduced resource fragmentation, and improved system utilization as can be seen by Figures 4 and 5.

From Figures 4 and 5 it can be observed that the number of requests accepted and utilization increased in case of the proposed algorithm in comparison to the existing algorithm.

### 5.2. Simulation Setup

To setup in large scale and evaluate performance metrics, a simulated environment is used. We expanded the CloudSim toolkit [22] to simulate the proposed cloud architecture and performed our experiments. The CloudSim toolkit supports both the system and behavior modeling of cloud system components, such as datacenters, virtual machines, and provisioning policies.

**Figure 6:**
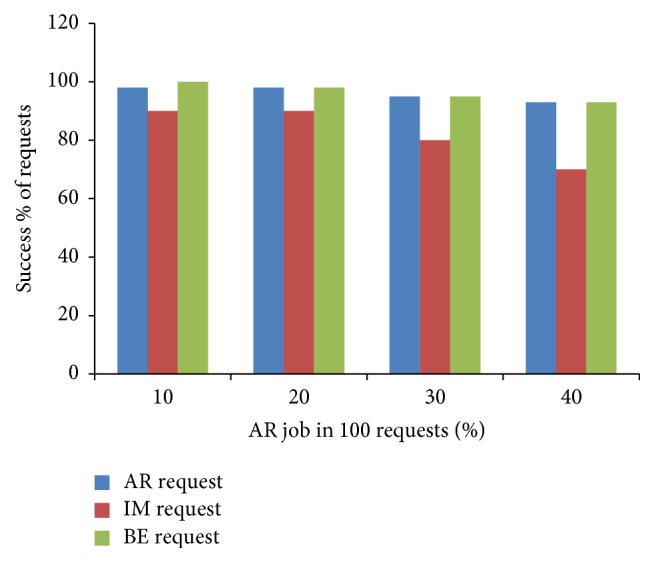
Evaluation of AR job requests.

The implementation has been accomplished by modifying the original source code of the simulator that was written in Java language. We have incorporated the proposed data structure in the modified simulation environment. Furthermore, RAL and PL are prepared and stored for processing query using MYSQL extension. The number of AR job and average duration of AR job highly influences the scheduling decision which, in turn, affects the successful execution of the submitted requests [13]. In order to find the percentage of AR job in our workload, we conducted an experiment where the percentage of AR request varied to observe the effect of different percentage of AR jobs in a workload. We have taken a total of 100 requests, which contains a mix of three types of requests (AR, IM, and BE) and the success percentage of these sets is plotted as shown in Figure 6. From Figure 6, we find that the success rate drastically reduced for IM request when more AR requests are present in the workload due to the unavailability of the resource. Hence we consider the number of AR jobs in our workload submitted list as 20% for further evaluation of other metrics. We considered the simulated workload trace given as [23] having AR and BE mode requests. An additional set of IM jobs are interleaved in between to generate the mix of the three types of requests randomly. From that we took 3 sets of 1000 jobs and evaluated for result analysis. Values assigned for simulation and host configuration are given in Tables 1 and 2. These parameters are kept constant at these values between different runs while comparing the results.

In order to show the effectiveness of our algorithm, we have compared the performance of the proposed algorithm with V-MCT [9] and priority scheduling [10] algorithms. V-MCT is minimum completion time algorithm without preemption and priority algorithm is preemptive algorithm. The same dataset is used to compare the results for all three algorithms. The compared algorithms methods are given below.

In V-MCT algorithm, the estimated total processing time (ETPT) of a job is calculated and the job is allocated to a VM which is available, where ETPT of *i*th job on *j*th host is calculated as (12)

(13) $$\begin{array}{c} \text{ETPT}\left[\;i,j\;\right]=\overset{w}{\underset{i=1}{\sum }}\frac{S_{i}}{b_{k}}+\underset{\left(i\in w,j\in y\right)}{\max}\left(\;\text{CT}\left[\;i,j\;\right]\;\right). \end{array}$$

ETPT value is the summation of time taken to transfer a job to a VM which is based on the ratio of the actual file size of the job, available bandwidth and completion time of a VM on a host.

In priority algorithm, each job requests a resource with a determined priority. The priority of each job is compared with other jobs separately

(14) $$\begin{array}{c} {pg}^{if}=\left\{\begin{array}{cc} \frac{1}{{pg}^{if}}, & i\neq f,\\ 1, & i=f. \end{array}\right. \end{array}$$

In (14), *pg* denotes a matrix with *m* rows and *m* columns. This matrix is a comparison matrix. For each of the comparison matrices, there is a need to compute a priority vector (vector of weights) for scheduling. Using iterative methods, priority vector (PV) (vector of weights) can be calculated. From that PV, the maximum element is selected to allocate a suitable resource.

### 5.3. Performance Metrics

Various performance metrics were taken into consideration in order to measure and evaluate the selected job scheduling algorithms. These metrics include the success rate, resource utilization, makespan, and total completion time.

*Success Rate*. The success rate is the ratio of number of jobs executed successfully to the total number of jobs submitted.

*Makespan*. The makespan represents the maximum finishing time among all received jobs per time. This parameter shows the quality of job assignment to resources from the execution time perspective

(15) $$\begin{array}{c} \text{Makespan}=\mathrm{Max}\left\{\;{FT}_{j}\;|\;\forall j\in JQ\;\right\}, \end{array}$$

where FT_*j*_ = the finishing time of job *j*. *j* = job from the list of jobs. 

*Resource Utilization U*. Let *C*_used_ be the used host capacity in terms of cores and *C* the total number of cores of a host; then

(16) $$\begin{array}{c} U=\frac{\sum_{t=1}^{m}\left(\sum_{j=1}^{J}{Cj}_{\left(\text{used}\right)t}/\sum_{t=1}^{m}C_{t}\right)}{m}. \end{array}$$

**Figure 7:**
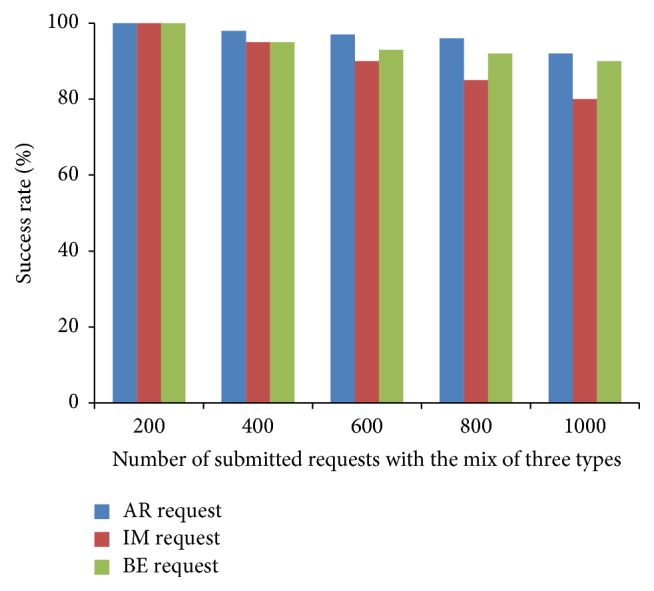
Success rate of proposed algorithm.

**Figure 8:**
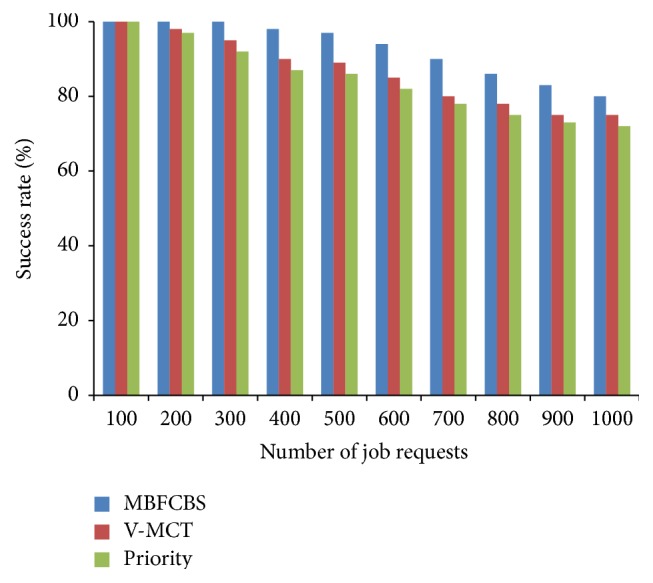
Success rate of scheduling algorithms.

**Figure 9 fig9:**
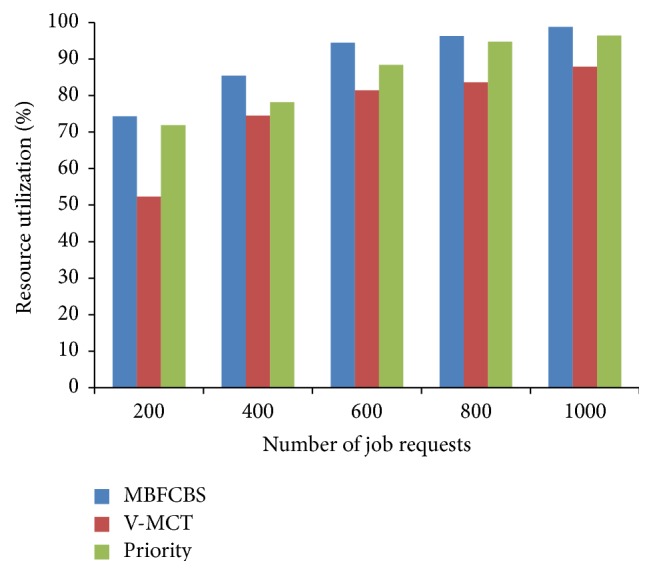
Resource utilization in percentage.

**Figure 10 fig10:**
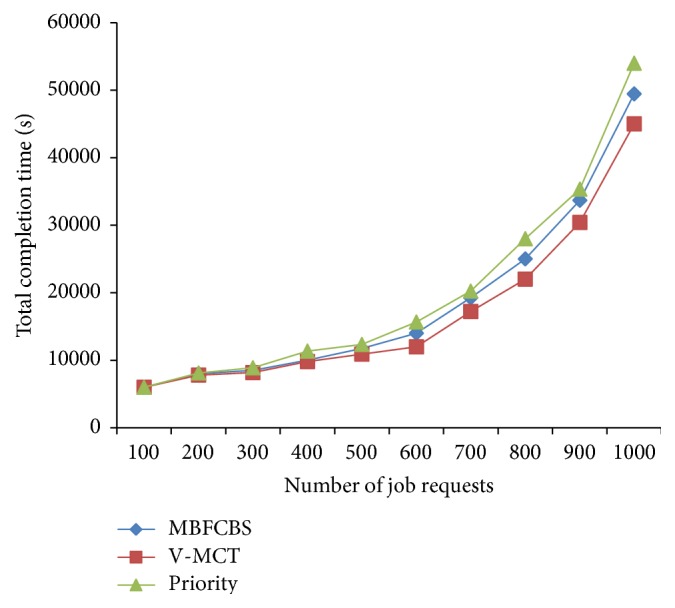
Comparison of total completion time.

**Figure 11:**
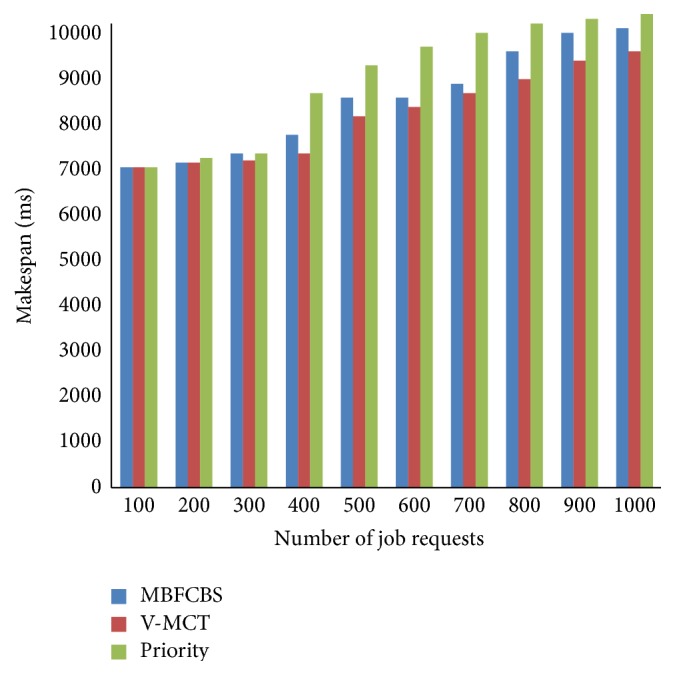
Makespan of scheduling algorithms.

From Figure 7, it can be observed that our algorithm gives high success rate to AR request and ensures a guaranteed service to AR request than IM request. IM requests get accepted when the resource is available, otherwise rejected. BE jobs are kept in the queue till execution and utilize the resources when it is free or idle. Therefore, the success rate of BE jobs is greater than IM request. The other scheduling metrics are compared and shown in Figures 8–11. From all the above results, we conclude that MBFCBS has achieved the highest success rate and utilization in all cases compared to the other algorithms. This is due to the fact that the MBFCBS algorithm attempts to select the most suitable VM that can rapidly respond and execute the given job. We observe from Figure 11 that makespan time and total completion time of MBFCBS are higher than that of V-MCT when the number of jobs increases. This is attributed to the fact that when more requests are submitted, MBFCBS preempts more BE jobs and puts these for backfilling later. This increases the average completion time of the over job requests, which also results in higher makespan. V-MCT algorithm performed better when more number of requests arrives compared to the proposed algorithm since V-MCT does not consider the preemption of BE jobs and arbitrarily chooses any finishing VM to assign the job. But V-MCT algorithm delays the other types of jobs to execute if any batch type of job is assigned on the VM. Since V-MCT algorithm does not support preemption of job requests. Hence, the success rate and throughput decrease which results in more failed job requests.

From the results we observe that the priority algorithm performs the worst among all algorithms considered with respect to makespan, success rate, and utilization. This is because the priority algorithm attempts to pick a host from a computed available vector list of hosts. For each job it computes a priority vector where less priority jobs are preempted. This leads to the starvation and failure of jobs having less priority. Also, computing priority and accessibility vectors take time, which further contribute to the increased makespan. MBFCBS shows improvement over the other two algorithms in terms of success rate and resource utilization since it takes the advantage of preemption and earliest available resources to achieve better results. The preemption of the job is also reduced through the flag to avoid starvation and due to the type classification of requests, these preempted jobs can be executed till the resource is free or reserved for advanced reservation. In that way, the resources are effectively utilized to the maximum which give higher utilization than the other two algorithms.

## 6. Conclusion

In this paper, we present cloud scheduling using proposed MBFCBS algorithm. This paper explored the problem of job (VM) placement in cloud providers' datacenter. Our original contribution consists of MBFCBS algorithm for scheduling that maximizes the resource utilization and improved success rate with the usage of an efficient data structure for resource monitoring and lookup mechanism. We investigated preemption as a way to increase resource utilization in datacenters, where some requests have preemptive priority over the others. Our proposed algorithm is based on linear integer programming model. Extensive experimentations brought forward promising results about the performance of the proposed algorithm along with the data structure in a cloud datacenter environment. Further investigation could be in the direction of the utility of this algorithm in other cloud scenarios such as including deadline sensitive request type and in a federated cloud.

## Figures and Tables

**Algorithm 1:**
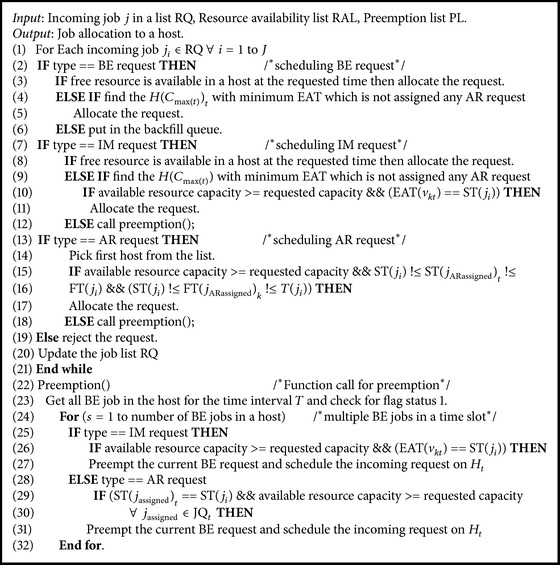
Modified best fit with capacity based scheduling (MBFCBS).

**Table 1:** Values assigned in simulation.

Specification	Value
Number of hosts	50
Number of cores (PEs)	6
Number of requests	100–1000, mixed equally with all three types of requests and randomly inserted

**Table 2:** Host configuration with VM type.

VM type	Host configuration				
	MIPS	Storage size	RAM	Bandwidth	PEs (cores)
Small	1000	104960	1920	1000	1
Medium	5000	419840	3840	1000	2
Large	10000	870400	7680	1000	4

## Notations Used in the System

RQ: Incoming request queue in CMS
JQ_*t*_: Job queue length of a host *t*
*H*: Host (server) in a datacenter
*C*_*t*_: Total number of cores in a host
NC(*j*_*i*_): Number of cores required by a job request (VM specification)
Mem_*t*_: Total memory of a host
RM(*j*_*i*_): Required memory of a request
CP_*t*_: Capacity of a host in terms of total number of cores and total memory
CP_limit_: Maximum capacity limit of a host to have *n* number of VMs at a time.
